# Intraoperative change of lactate level is associated with postoperative outcomes in pediatric cardiac surgery patients: retrospective observational study

**DOI:** 10.1186/s12871-015-0007-y

**Published:** 2015-03-08

**Authors:** Tomoyuki Kanazawa, Moritoki Egi, Kazuyoshi Shimizu, Yuichiro Toda, Tatsuo Iwasaki, Hiroshi Morimatsu

**Affiliations:** Department of Anesthesiology and Resuscitology, Okayama University Hospital, 2-5-1 Shikatachou, Kitaku, Okayama, Okayama 700-8525 Japan

**Keywords:** Intraoperative, Lactate, Pediatric cardiac surgery, Cardiopulmonary bypass, Outcome

## Abstract

**Background:**

A change of serum lactate concentrations appeared to be useful for predicting outcomes in various acute ill settings. However, there is little information on intraoperative change of lactate level in pediatric cardiac surgery patients.

**Methods:**

We conducted a retrospective observational study of 459 children who received pediatric cardiac surgery to determine the association between change of lactate level after cardiopulmonary bypass (CPB) and patient prognosis (length of ICU stay and incidence of postoperative serious adverse events (SAEs)). We defined change of lactate level after CPB (LAC⊿) as (final lactate level measurement in the operating room) – (lactate level measured at the end of CPB). To study the independent association of LAC⊿ with length of ICU stay, we used linear regression model.

**Results:**

There were 1145 lactate measurements after CPB in this study cohort. After weaning from CPB, the serum lactate levels significantly increased from 2.1 mmol/L to 2.5 mmol/L (p < 0.001). Patients with higher LAC⊿ had significantly longer stay in ICU (p = 0.017) and higher incidence of SAEs (p = 0.002). In multivariate linear regression analysis, higher LAC⊿ showed a significant independent association with longer length of ICU stay.

**Conclusions:**

Increased lactate level after CPB was associated with the longer duration of ICU stay and increased risk of postoperative SAEs in pediatric cardiac surgery patients. Future studies should be conducted to determine the clinical utility of intraoperative trend of lactate levels.

**Electronic supplementary material:**

The online version of this article (doi:10.1186/s12871-015-0007-y) contains supplementary material, which is available to authorized users.

## Background

Hyperlactatemia observed in postoperative intensive care unit were shown to have predictability for worse outcomes in pediatric patients undergoing cardiac surgery [1-6]. Early prediction of worse outcomes might be useful to improve outcome by widening the therapeutic time window or facilitating early intervention. In this regards, it could be desired to assess whether intraoperative lactate indices might have significant association with postoperative worse outcomes in pediatric patients undergoing cardiac surgery.

The increase of blood lactate level would be caused by increasing lactate production, decreasing lactate clearance at liver, kidney and other organs, or both simultaneously [7]. The change of lactate concentration appears to be useful for predicting outcomes in various acute ill settings [8-14]. Furthermore, one study has suggested that interventions aimed at targeting a lactate reduction in the critically ill with an abnormal lactate level may reduce organ failure and increase survival [15]. However, the change of lactate levels immediately following weaning from cardiopulmonary bypass (CPB) and its association with postoperative outcomes has been poorly investigated in pediatric patients undergoing cardiac surgery.

Accordingly, to test the association between lactate change after CPB and patients’ outcome, we conducted a retrospective observational study. We hypothesized that larger change of lactate level after CPB would be associated with increased the duration of ICU stay and the incidence of postoperative serious adverse events (SAEs) in pediatric patients who have undergone pediatric cardiac surgery.

## Methods

### Design

This is a retrospective observational study, for which the institutional review board of Okayama university waived the need for obtaining informed consent and approved its submission for publication.

### Study population and data sources

This study was conducted in a tertiary teaching hospital that had 865 beds in the hospital and 8 beds in the pediatric cardiac ICU. We studied consecutive pediatric patients who underwent pediatric cardiac surgery during the period from January 1, 2007 to May 31, 2011. Inclusion criteria for the current study were age <18 years, undergone pediatric cardiac surgery that required CPB, and with at least two lactate measurements after the end of CPB.

Data on age, gender, the lowest temperature during CPB and the duration of CPB were obtained from the patients’ database, which had been prospectively collected by trained physicians. Coding for type of surgery was done by means of The Risk-Adjusted Classification for Congenital Heart Surgery Version 1 (RACHS-1) category [16]. We also obtained whether the patient received epinephrine administration after CPB or not.

### Lactate concentration measurements and lactate change after CPB

All blood lactate concentrations were measured by an arterial blood gas analyzer (ABL 800, Radiometer Co., Copenhagen, Denmark). The lactate data for this study were corrected to a pH of 7.40. The lactate data reported here were stored and retrieved electronically. Blood samples were collected in standard pre-prepared heparinized blood gas syringes. The analyzer measured whole blood samples at 37 degrees. Trained physicians performed all blood gas analyses. Laboratory in the hospital complied with standards of the National Association of Testing Authorities. During the study period, arterial blood gas analyses were performed according to the decision of the anesthetic specialist. Lactate ringer’s solution was not used for fluid therapy during perioperative management.

We calculated delta lactate after CPB (LAC⊿) by the following: (last measurement of lactate level in the operating room(Lac_Last_)) – (lactate level measured at the end of CPB(Lac_First_)).

### Primary and secondary outcomes

Primary outcome was the duration of ICU stay. Secondary outcome was the incidence of at least one of SAEs. We defined SAEs including cardiac arrest, the requirement of extracorporeal membrane oxygenation (ECMO), and death during intensive care unit. All information was prospectively collected by trained physicians and stored electronically.

### Statistical analysis

Data are presented as percentages (n) or as median (25% quartile, 75% quartile). We first assessed the association between LAC⊿ and the duration of ICU stay. Since its relationship may not be linear in nature, we separated patients into four subgroups according to each 1 mmol/L of LAC⊿. This categorization was planned prior to analysis. We performed multivariate linear regression analysis with adjustment for the following covariates: age, gender, RACHS-1, the lowest temperature during CPB, the duration of CPB, the use of epinephrine after operation, the duration between Lac_FIRST_ and Lac_Last_, Lac_First_ and subgroups of LAC⊿. The dependent variable was the duration of ICU stay.

To assess the influence of the duration between Lac_FIRST_ and Lac_Last_, we further calculate the time weighted change of lactate level after CPB (LAC⊿tw); LAC⊿divided by the duration between Lac_FIRST_ and Lac_Last_. We separated our patients into 4 groups according to quartile of LAC⊿tw (group1; 1^st^ quartile, group2; 2^nd^ quartile, group3; 3^rd^ quartile and 4^th^ quartile; group4), and performed same analysis with LAC⊿.

P values of less than 0.05 were considered statistically significant. All statistical analyses were performed using commercially available statistical software (SPSS 19.0, SPSS Inc., Chicago, IL). Data was reported in accordance with the Strengthening the Reporting of Observational Studies in Epidemiology (STROBE) guidelines [17].

## Results

### Study flow

During study period, there were 459 pediatric patients who received pediatric cardiac surgery required CPB and had at least two lactate measurements after the end of CPB. There was no patient with missing values.

### Patients’ demographics and outcomes

Among study cohort, the median age was 14 months and median RACHS-1 category was 3. The median CPB time was 91 minutes. ICU stay in our cohort was a median of 6 days. There are 11 patients (2.4%) who had at least one SAEs; 2 patients with cardiac arrests, 5 patients required ECMO postoperatively and 7 patients died in ICU.

### Lactate measurements after CPB

There were 1145 lactate measurements after CPB in this study cohort. The median LAC⊿ was 0.3 mmol/L. After weaning from CPB, the serum lactate levels were significantly increased from 2.1 mmol/L to 2.5 mmol/L (p < 0.001) (Table 1). The duration between Lac_FIRST_ and Lac_Last_ was median of 62 minutes.

**Table 1:** **Patient’s demographics**

Variables	median (IQR) or n(%)
Age (months old)	14 (3, 45)
Gender (male)	236/459(52.7%)
RACHS-1	3(2, 3)
Lowest temperature during CPB (°C)	32 (28,32)
CPB time, (min)	91 (66, 128)
The use of epinephrine after weaning from CPB	60/459 (13.1%)
ICU stay (day)	6 (3, 8)
The duration between Lac_FIRST_ and Lac_Last_ (min)	62 (37, 87)
Lac_First_(mmol/L)	2.1 (1.3, 3.2)
Lac_Last_ (mmol/L)	2.5 (1.6, 3.8)
LAC⊿ (mmol/L)	0.3 (0, 1.0)

RACHS-1: Risk-Adjusted classification for Congenital Heart Surgery Version 1.

CPB: cardiopulmonary bypass.

ICU: intensive care unit.

Lac_FIRST_: lactate level measured at the end of cardiopulmonary bypass.

Lac_LAST_: lactate level measured at the end of operation.

LAC⊿: change of lactate levels after weaning from cardiopulmonary bypass.

### Univariate association of LAC⊿ with outcomes

Table 2 shows the duration of ICU stay among subgroups according to LAC⊿. The population of each groups were 101 (LAC⊿≦0 mmol/L), 235 (LAC⊿ 0-1mmol/L), 86 (LAC⊿ 1-2mmol/L) and 37(LAC⊿ >2mmol/L).The duration of ICU stay significantly differed among subgroups (p = 0.017) and increased according to increase of LAC⊿. To assess the influence of age on this association, we separated patients into two groups according to median of ages and performed same analysis in both groups. There was significant association between LAC⊿ and length of ICU stay both in younger group (median of 3 months old, p < 0.001) and elder group (median of 3 years old , p = 0.016).

**Table 2:** **The association of the duration of ICU stay with delta change of lactate levels according to each 1 mmol/L**

Delta change of lactate levels	≦0	0-1	1-2	2<	P-value
ICU stay median (IQR)	5 (3, 7)	5 (3, 8)	6 (3, 11)	6.5 (4, 13)	0.017

ICU; intensive care unit.

IQR; interquartile range.

**Figure 1:**
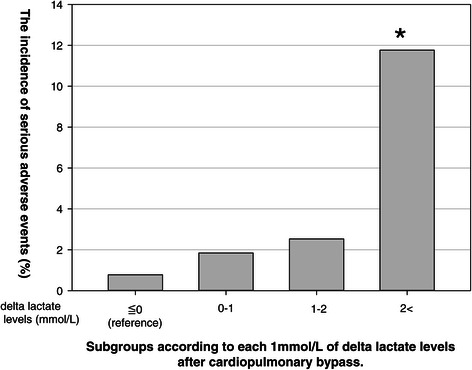
**The incidence of serious adverse events among subgroups according to each 1mmol/L of delta lactate levels after cardiopulmonary bypass.** This figure shows the incidence of serious adverse events among subgroups according to delta lactate levels after cardiopulmonary bypass. The incidence of serious adverse events was significantly differed among subgroups (p = 0.002) and increased when delta lactate level was >2 mmol/L in compared with those with >0 mmol/L.

Figure 1 shows the incidence of SAEs among subgroups according to LAC⊿. The incidence of SAEs significantly differed among subgroups (p = 0.002) and increased when LAC⊿ was >2 mmol/L.

### Multivariate analysis

In our multivariate linear regression model including possible confounders, LAC⊿ >2mmol/L was independently associated with increased the duration of ICU stay (p = 0.028) (Table 3). In this analysis, Lac_FIRST_ was not independently associated with the duration of ICU stay (p = 0.41).

**Table 3:** **Multivariate analysis for duration of ICU stay**

	Coefficient	p-value
Age (months old)	0.007	0.54
Gender (male)	−0.4	0.85
RACHS-1	2.5	0.049
Lowest temperature during CPB (°C)	−0.6	0.08
CPB time (min)	0.006	0.17
The use of epinephrine after weaning from CPB	15.1	<0.001
The duration between Lac_FIRST_ and Lac_Last_ (min)	−0.24	0.65
Lac_FIRST_ (mmol/L)	0.7	0.42
LAC⊿≦0 mmol/L	reference	
LAC⊿ 0-1mmol/L (vs. LAC⊿≦0 mmol/L)	2.1	0.42
LAC⊿ 1-2mmol/L (vs. LAC⊿≦0 mmol/L)	0.8	0.81
LAC⊿ >2mmol/L (vs. LAC⊿≦0 mmol/L)	10.3	0.028

ICU: intensive care unit.

RACHS-1: Risk-Adjusted classification for Congenital Heart Surgery Version 1.

CPB: cardiopulmonary bypass.

Lac_FIRST_: lactate level measured at the end of cardiopulmonary bypass.

Lac_LAST_: last measurement of lactate level in the operating room.

LAC⊿:change of lactate level after cardiopulmonary bypass.

### The time weighted change of lactate level after CPB

Length of ICU stay was significantly different among 4 groups according to the quartile of LAC⊿tw (p = 0.049). The higher LAC⊿tw was associated with increased length of ICU stay. We performed the another multivariate linear regression model using subgroups of LAC⊿tw (Table 4). Group 4 (the larger LAC⊿tw) was independently associated with increased the duration of ICU stay (p = 0.048) (Table 4).

**Table 4:** **Multivariate analysis for duration of ICU stay with the subgroups according to the interquartile of time weighted delta lactate change**

	Coefficient	p-value
Age (month)	0.01	0.39
Gender (male)	0.20	0.93
RACHS-1	1.75	0.19
Lowest temperature during CPB (°C)	−0.33	0. 31
CPB time (min)	0.015	0. 55
The use of epinephrine after weaning from CPB	16.8	<0.001
Lac_FIRST_ (mmol/L)	1.17	0.17
Group1(1^st^ quartile of LAC⊿tw)	reference	
Group2 (2^nd^ quartile of LAC⊿tw) (vs. Group1)	2.60	0.39
Group3 (3^rd^ quartile of LAC⊿tw) (vs. Group1)	0.16	0.96
Group4 (4^th^ quartile of LAC⊿tw) (vs. Group1)	6.14	0.048

ICU: intensive care unit.

RACHS-1: Risk-Adjusted classification for Congenital Heart Surgery Version 1.

CPB: cardiopulmonary bypass.

Lac_FIRST_: lactate level measured at the end of cardiopulmonary bypass.

LAC⊿tw: time weighted change of lactate level after cardiopulmonary bypass.

## Discussion

### Key finding

We performed a retrospective study to evaluate the usefulness of intraoperative lactate change after CPB for predicting worse postoperative outcomes in pediatric cardiac surgery patients. In our study, the duration of ICU stay was significantly increased according to higher LAC⊿. LAC⊿ >2 mmol/L was also associated with the increased incidence of SAEs. Even after adjusting for relevant confounders, LAC⊿ was independently associated with increased duration of ICU stay. This finding was consisted even when we used the time weighted change of lactate level after CPB.

### Comparison with prior studies

While several studies have demonstrated that higher blood lactate levels at weaning from CPB [18], chest closure [18] and end of the operation [19,20], and maximum lactate level during the operation [21] were associated with worse outcomes in pediatric cardiac surgery patients, there has been a few report on the predictability of intraoperative change of lactate concentration for postoperative outcomes.

Munoz et al. have shown that increased lactate concentration during CPB (defined as the difference between baseline and the first lactate value obtained after CPB) was associated with the risk of postoperative SAEs [1] in 174 pediatric cardiac surgery patients who required CPB. In their multivariate logistic analysis, lactate change during CPB tended to be independently associated with mortality (p = 0.13) and risk of complication (p = 0.06). Park et al have shown that the lactate change from CPB weaning to chest closure was significantly associated with major adverse events [18]. However, they did not assess its independent associations with patients’ outcomes.

To our knowledge, this is the first study to assess the independent predictability of intraoperative lactate change after CPB for worse outcomes in pediatric cardiac surgery patients. Although results did not conflict with the prior study, further studies are required to confirm the clinical utility of this measure.

### Interpretation

It might be relevant to find biomarkers which can identify patients who are at higher risk of worse outcomes. Although our findings have a similarly with prior studies to show that the significant association of the increase of lactate concentration with increased risk of worse outcomes in other settings [8-10,12,13], there were still few studies to assess the association between intraoperative lactate changes and outcomes in pediatric cardiac surgery patients.

In compared with the lactate concentration in postoperative ICU, intraoperative lactate change had advantage to promote the re-assess the quality of operation, cardiac function, circulatory blood volume and ventilator setting in operation room. This fact gives physicians the time windows to early intervention including administration of inotrope, adjustment of circulatory blood volume and alternation of ventilator settings. We should note that such an association existed even after adjusting the value of Lac_FIRST_ and the duration between Lac_FIRST_ and Lac_Last_. This may suggest that irrespective of initial lactate value after CPB and duration between measurements, the lactate change after CPB was useful for prediction of worse outcomes.

### Limitation

Our study has several limitations. First, it is retrospective in design and thus potentially subject to systematic error and bias. However, the clinical and electronic data were collected prospectively, are numerical in nature and were measured independently, and were thus not amenable to unintended manipulation.

Second, the study was conducted in only one hospital. The findings might be different for studies conducted in other hospitals or in other countries with different styles of management. However, it should be noted that there has been no multicenter study in which the association of lactate indices with outcomes in pediatric cardiac surgery patients was assessed. A multicenter study should be conducted in the future to refute or confirm our findings.

Third, there is no protocol for lactate measurements, and the duration of post CPB (between end of CPB and surgery) varied among patients. Such a time distribution of LAC⊿ might affect our findings. However, we performed multivariate analysis adjusting the duration between Lac_FIRST_ and Lac_Last_ and found that higher LAC⊿ had independent association with increased the length of ICU stay. Furthermore, we calculated the time weighted change of lactate level after CPB and also found that the higher LAC⊿tw was independently associated with increased length of ICU stay.

Fourth, in current study, we used pre-planed subgroups according to each 1 mmol/L of LAC⊿. Thus, our study could not determine the threshold of LAC⊿ to maximize its predictability of worse outcomes. Accordingly, we performed post-hoc analysis to determine the threshold of LAC⊿ to have larger area under the receiver operator characteristic curve (AUROC curve). Then, we found that LAC≧1.6mmol/L had largest AUROC curve for the length of ICU stay and the incidence of SAEs (Additional files 1 and 2). As this is post hoc analysis, future study is necessary to address the threshold of intraoperative change of lactate level for greater predictability of postoperative outcomes.

Fifth, we included patients with wide range of age. As age may influence the metabolic rate and oxygen tissue oxygen requirements after surgery, the association with LAC⊿ and patients’ outcomes may varied in different ages. We found that there was significant association between LAC⊿ and length of ICU stay both in younger and elder patients. Nonetheless, we believed that future study is necessary to compare the magnitude of association between LAC⊿ and outcomes among patients with different ages.

Sixth, we included the 26 children required circulatory arrest during surgery. This fact may influence our findings. However, even after excluded these 26 patients, we found that the duration of ICU stay significantly increased according to each category of LAC⊿ (p = 0.017).

Finally, although we assessed the independent association of LAC⊿ with outcomes with adjustment for age, gender, lowest temp CPB, CPB time, RACHS-1 the duration between Lac_FIRST_ and Lac_Last_ and epinephrine use, there may be other confounders that influenced the association between LAC⊿ and outcomes. In this regards, future study should be collected on these possible confounders to avoid potential biases.

## Conclusions

In conclusion, increased lactate concentration after CPB was associated with risk of postoperative SAEs in pediatric cardiac surgery patients. Future studies should be conducted to confirm the potential utility of lactate change after CPB as a future therapeutic target or trigger.

## Additional files

Additional file 1: **This figure showed the area under the receiver operator characteristic curve for the length of ICU stay in every 0.2mmol/L of LAC⊿.** We found that largest the area under the receiver operator characteristic curve was seen when the threshold was 1.6mmol/L of LAC⊿. Additional file 2: **This figure showed the area under the receiver operator characteristic curve for the incidence of postoperative serious adverse events.** The Maximum (sensitivity + specificity-1) was see at LAC⊿ of 1.6mmol/L.

## Abbreviations

CPB: Cardiopulmonary bypass
SAEs: Serious adverse events
LAC⊿: Lactate level after cardiopulmonary bypass
RACHS-1: The Risk-Adjusted Classification for Congenital Heart Surgery Version 1
Lac_Last_: Last measurement of lactate level in the operating room
Lac_First_: Lactate level measured at the end of cardiopulmonary bypass
ECMO: Extracorporeal membrane oxygenation
LAC⊿tw: The time weighted change of lactate level after cardiopulmonary bypass
AUROC curve: Area under the receiver operator characteristic curve
