# Supplementary material for: Intraoperative change of lactate level is associated with postoperative outcomes in pediatric cardiac surgery patients: retrospective observational study
Source: BMC Anesthesiol. 2015 Mar 8;15:29. doi: 10.1186/s12871-015-0007-y (PMC4354761; doi:10.1186/s12871-015-0007-y)
Supplement: Additional file 1: — This figure showed the area under the receiver operator characteristic curve for the length of ICU stay in every 0.2mmol/L of LAC⊿. We found that largest the area under the receiver operator characteristic curve was seen when the threshold was 1.6mmol/L of LAC⊿. [file 12871_2015_7_MOESM1_ESM.pptx]

## Slide 1
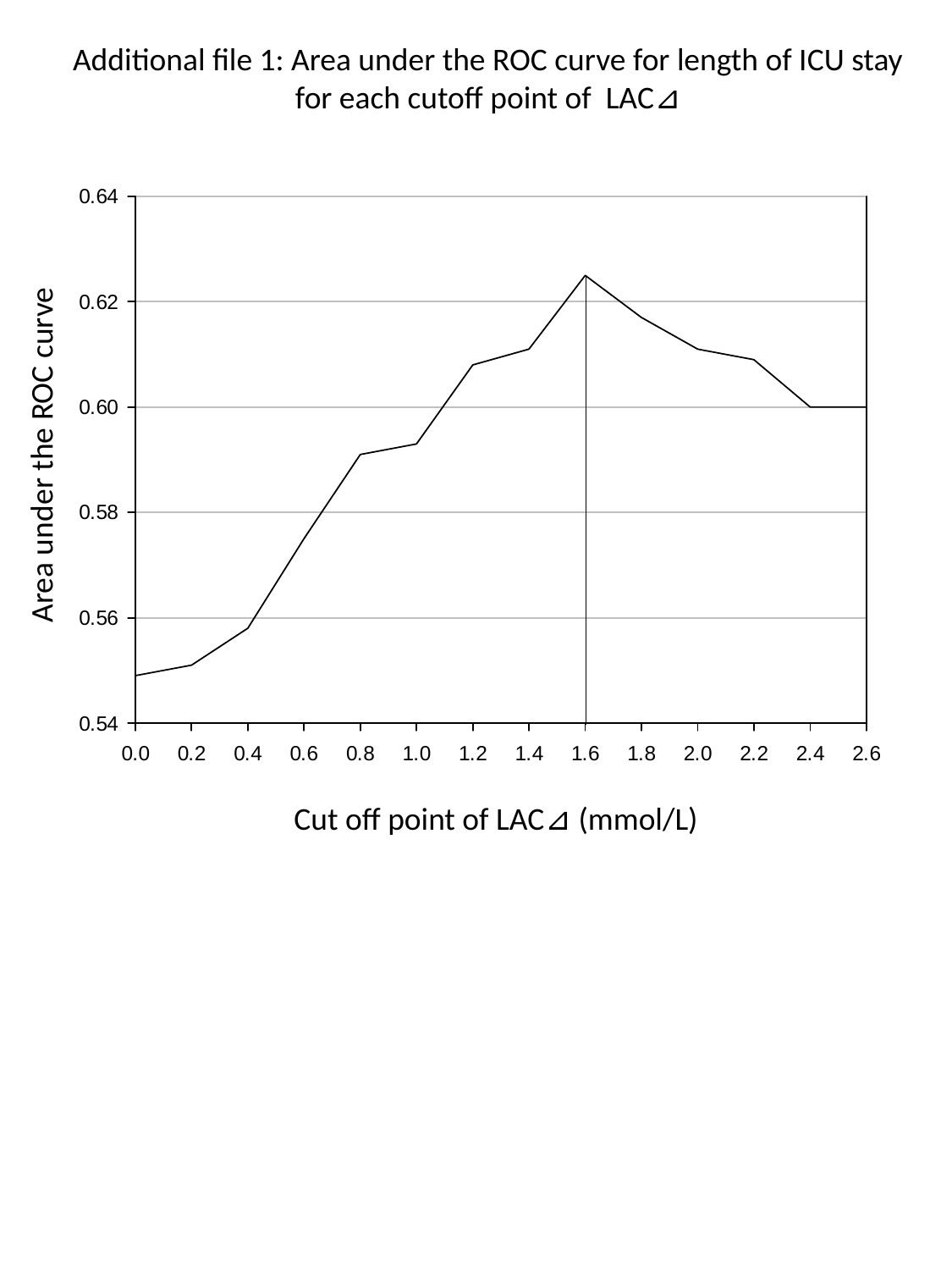

Additional file 1: Area under the ROC curve for length of ICU stay for each cutoff point of LAC⊿
Area under the ROC curve
Cut off point of LAC⊿ (mmol/L)
