# Supplementary figures and images for: Intraoperative change of lactate level is associated with postoperative outcomes in pediatric cardiac surgery patients: retrospective observational study
Source: BMC Anesthesiol. 2015 Mar 8;15:29. doi: 10.1186/s12871-015-0007-y (PMC4354761; doi:10.1186/s12871-015-0007-y)

## Slide 1
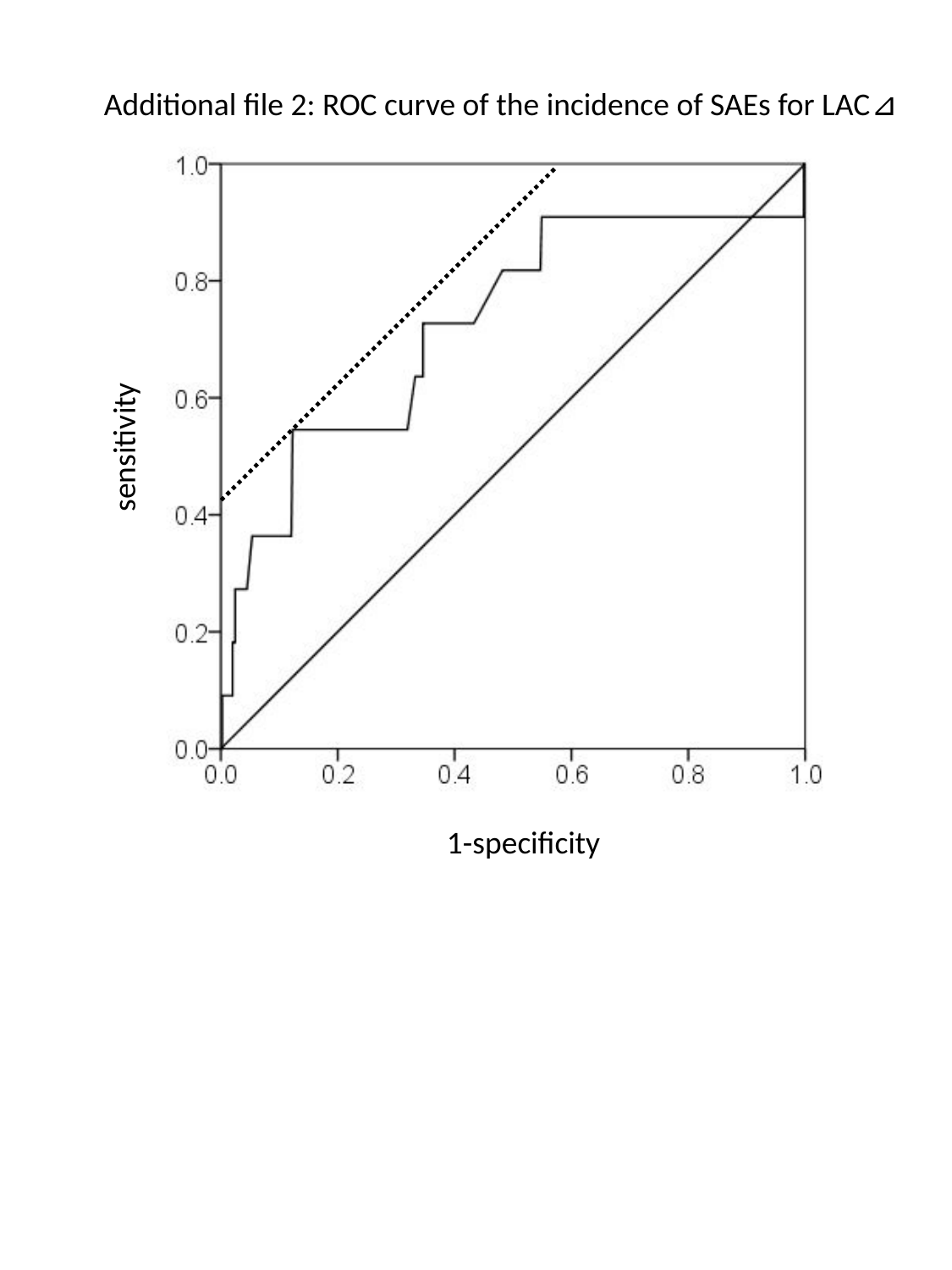

Additional file 2: ROC curve of the incidence of SAEs for LAC⊿
sensitivity
1-specificity

Supplement: Additional file 2: — This figure showed the area under the receiver operator characteristic curve for the incidence of postoperative serious adverse events. The Maximum (sensitivity + specificity-1) was see at LAC⊿ of 1.6mmol/L. [file 12871_2015_7_MOESM2_ESM.pptx]
